# Genetic diversity analysis of wild tea plant resources in Chongzuo based on phenotypic traits and SSR molecular markers

**DOI:** 10.1371/journal.pone.0337070

**Published:** 2025-11-18

**Authors:** Zongyou Wei, Feifei Wu, Hongyu Feng, Ziping Li, Qinhao Jian, Yunxiong Zhao, Baogui Liu, Jinting Li

**Affiliations:** Guangxi South Asian Tropical Agricultural Science Research Institute, Chongzuo, China; Government College University Faisalabad, PAKISTAN

## Abstract

To explore the genetic diversity of wild tea plants, the methods of the phenotypic traits and SSR molecular marker analysis were adopted to analyze the 40 samples of 7 wild tea populations in Chongzuo, Guangxi. The results showed that the coefficient of variation of 18 descriptive phenotypic traits ranged from 15.76% to 50.64%, and the diversity index ranged from 0.38 to 1.04. The coefficient of variation of the four numerical phenotypic traits ranged from 31.91% to 56.86%. The diversity index ranged from 1.70 to 1.93. Based on phenotypic trait clustering, the 40 tea plant samples were divided into three distinct groups. A total of 117 alleles (Na) were detected by 14 pairs of SSR primers. The polymorphism information content (PIC) of primers ranged from 0.25 to 0.87. The study showed that the optimal K value for population number, based on genetics, was 2. Meanwhile, the genetic distance among populations ranged from 0.24–0.74, the percentage of variation among populations was 20.46%. The genetic distance between the 40 tea plant resources ranged from 0.20–0.95, and the percentage of variation between individuals was 74.22%. When the individual genetic distance was 0.33, the 40 tea plant resources were divided into three groups. This study elucidates the genetic diversity of 40 wild tea plant accessions from seven populations in Chongzuo, Guangxi, which can provide a theoretical basis for breeding superior tea cultivars and conserving tea plant resources.

## 1. Introduction

Tea (*Camellia sinensis* [L.] O. Kuntze) is an evergreen plant belonging to the family Theaceae and the genus Camellia [1–3]. Through long-term evolution and widespread cultivation, it has developed numerous varieties and ecotypes. The species is naturally distributed across Southwest China, South China and northern parts of Southeast Asia. Chongzuo in Guangxi, located in southwest of China (21 ° 36 '−23 ° 22' N, 106 ° 33'] −108 ° 6 'E), has a long history of tea cultivation. This region harbors many wild tea plant populations and holds significant potential for future development [4]. As such, Chongzuo offers considerable value for research on the origin of tea plants, biodiversity, and breeding of superior cultivars [5].

Genetic diversity analysis plays a crucial role in the identification and evaluation of germplasm resources, aiding in the discovery and selection of superior germplasm [6]. Phenotypic trait analysis offers an intuitive, straightforward and cost-effective approach to assess genetic diversity in tea plants, helping to elucidate patterns of genetic variation based on observable characteristics. For instance, a study of 89 wild tea resources from Yingde revealed rich genetic diversity [7]. Similarly, research on 195 arbor-type tea germplasm resources in Guizhou Province showed considerable variation in the coefficient of variation among different numerical trait types, although the genetic diversity indices differed only slightly [8]. Phenotypic traits enable rapid identification of desirable characteristics, thereby improving breeding efficiency. However, these traits are easily influenced by environmental factors, and relying solely on phenotypic data may compromise the accuracy of genetic diversity assessments. In contrast, Molecular marker technology provides a more accurate and stable genetic diversity analysis.

SSR molecular marker technology is widely used in genetic diversity studies of tea plants due to its advantages of high polymorphism, good repeatability, co-dominant inheritance and abundance [9]. For example, this method was applied to analyze the genetic diversity and relationships among 45 Dali tea germplasm samples and 5 Pu ‘er tea germplasm samples from the Lancang River Basin. The results indicated that Dali tea germplasm resources exhibit moderate genetic diversity and richness, with classification correlating to geographical origin [10]. Similarly, in a study of wild tea germplasm from Liubao Town and Nandu Town in Wuzhou City, SSR markers revealed abundant genetic diversity and high utilization value of the local tea resources [3]. Currently, there are few reports on the genetic diversity of wild tea resources in Chongzuo area. Therefore, evaluating the wild tea resources in this region will facilitate the discovery, innovative utilization, and conservation of valuable germplasm. In this study, phenotypic traits and SSR markers were used to analyze the genetic diversity of 40 wild tea plant samples from seven populations in Chongzuo. Th findings aim to provide a theoretical basis for the identification, conservation and utilization of unique tea resources in Chongzuo and Guangxi, as well as to support future breeding efforts.

## 2. Materials and methods

### 2.1. Plant material

The 40 samples of wild tea plants were collected from Chongzuo, Guangxi. detailed information (Table 1) .

**Table 1:** Resource information of 40 tea plants in Chongzuo.

Population code	Distribution location	Population mane	elevation	Latitude and longitude	Material name	Number of material
QS	Qingshan Village	Qingshan Population	716.53	106°41′14″E 22°19′47″N	S1-S5.	5
RX	Raoxiu Village	Raoxiu Population	702.24	106°41′16″E 22°19′45″N	S6-S10.	5
CQ	Changqiao Village	Changqiao Population	650.61	107°8′10″E 22°1′14″N	S11-S20.	10
XL	Xinlin Village	Xinlin Population	308.23	107°42′31″E 22°52′25″N	S21-S25.	5
LT	Liutou Village	Liutou Population	540.22	107°50′32″E 22°13′13″N	S26-S30.	5
NN	Nanei Village	Nanei Population	440.51	107°41′29″E 22°51′1″N	S31-S35.	5
JB	Jiangbian Village	Jiangbian Population	340.50	107°50′30″E 22°13′15″N	S35-S40.	5

### 2.2. Observation method of phenotypic traits of tea plant

The phenotypic traits of 40 tea samples were observed and statistically counted (Table 2), referring to the “Specification and Data Standards for the Description of Tea Germplasm Resources” [11]. The descriptive phenotypic traits were repeatedly observed 10 times, and the numerical phenotypic traits were repeatedly measured 3 times. Finally, the result of this research was the average value of the three measurements.

**Table 2:** Trait codes and statistical methods.

traits	Determination method and assignment standard
Tree type	1 Shrub; 2 Small tree; 3 Arbor
Tree posture	1 Erect; 2 Semi-spreading; 3 Spreading
Branch density	1 Dense; 2 Medium; 3 Sparse
Bud and leaf color	1 Opalescent white; 2 Yellow-green; 3 Light green; 4 Medium green; 5 Purplish-green
Bud and leaf pubescence	1 Glabrous; 2 Sparse; 3 Medium; 4 Dense; 5 Very dense
Leaf blade shape	1 Suborbicular; 2 Ovate; 3 Elliptic; 4 Oblong-elliptic; 5 Lanceolate
Leaf blade color	1 Yellow-green; 2 Light green; 3 Medium green; 4 Dark green
Leaf surface relief	1 Flat; 2 Slightly raised; 3 Strongly raised
Leaf blade curvature	1 Incurved; 2 Flat; 3 Slightly recurved
Leaf blade texture	1 Soft; 2 Medium; 3 Hard
Serration sharpness	1 Sharp; 2 Medium; 3 Blunt
Serration density	1 Sparse; 2 Medium; 3 Dense
Serration depth	1 Shallow; 2 Medium; 3 Deep
Leaf base shape	1 Cuneate; 2 Subrounded
Leaf apex shape	1 Abruptly acute; 2 Acuminate; 3 Obtuse; 4 Rounded
Leaf margin undulation	1 Flat; 2 Slightly undulate; 3 Undulate
Leaf attitude	1 Erect; 2 Semi-erect; 3 Horizontal; 4 Pendulous
Leaf size	1 Small-leaf; 2 Medium-leaf; 3 Large-leaf; 4 Extra-large-leaf
Leaf length	Straight ruler measurement, accurate to 0.1 cm.
Leaf width	Straight ruler measurement, accurate to 0.1 cm.
Length-width ratio	Leaf length/ leaf width.
Leaf area	Leaf length (cm) × leaf width (cm) × 0.7.

### 2.3. SSR primer information

The primer sequences were based on (‘*Camellia sinensis* (L.) O. Kuntze cv. Shuchazao’) genome design and selection of some of the reported SSR primers for tea plants, detailed information were shown in Table 3 [12–13], and the primers were synthesized by Wuhan Tianyi Huayu Gene Technology Co., Ltd.

**Table 3:** Sequences information of 14 pairs of SSR prime.

Primer name	(5’ → 3’) Primer sequences	Repeat motif	Tm (°C)	Fragment (bp)
CSK014	F-TTCGACCCGAAAGCGAAAAA R- GAGCTCCCACAAGGCTTAGG	(TATC)_8_	58	128
CSK065	F-TTGCCACCATTTTCCAAGCC R-TGGCTTATCGATTGTGGCCA	(ATT)_20_	59	274
CsFM1715	F-CGACCTGGTTTTGCATTTTT R-CCACGCTTGAGACAGATTGA	(TG)_14_	56	165
CsFM1051	F-AACCCATTTCGTCTTTGTGC R-AGAATCAACAACACCCTGGC	(TTG)_8_	57	124
CsFM1068	F-CAGGCCTTCGTTTTCTTTTG R-TTCCTCATCTTCTGTCTCCCTC	(TTC)_11_	56	212
CsFM1058	F-CCCCTCCATCTATCTCCCTC R-GGACTGTTGTTGGGGAAGAA	(TTC)_9_	57	162
CsFM1599	F-GGCCCTGTTTTTACACTCCA R-GATTGGTTTCTGGTTCGCAT	(CCTTC)_5_	56	167
CsFM1828	F-CAGCCGAGTCAACCGTAACT R-CTCTGATGTCGATCTGCCTG	(CAT)_7_	57	116
CsFM1696	F-TCCAAAAGATTCGGTTCGTC R-TCGATATTTTCCTTCCGTCG	(ACC)_8_	55	249
478	F-CAACACCACCAACAAGA R-GATATGAGATCCGTCCC	(AAAGG)_4_	51	110
CsFM1158	F-CGTGCCTGCATTGCTAATAA R-GTACCAGTAACTGCGGGGAA	(CACACC)_6_	56	220
SSR1	F-TTAAGCAAAGAAGTCGCG R-CTAAAATCTCCACTCAGCT	(CA)_8_	52	153
A5	F-ATGCTATTCCTCCGTCTC R-TTCAGGGATTGGTTTCTC	(AG)_12_	54	119
234	F-CTCCGATTACTTTCTTCC R-GCAGGTTAGCGGTGGTTA	(AAAAAG)_3_	53	151

### 2.4. Sequence amplification

Genomic DNA was extracted from tea samples using a plant tissue genomic DNA extraction kit. The quality and concentration of DNA were assessed using a micro-volume nucleic acid protein analyzer, and the purity was evaluated by 1% agarose gel electrophoresis. And then the qualified DNA was stored at −20°C for future use. The PCR amplification system included 500 ng of template DNA, 1 μL each of forward and reverse primers, 12.5 μL of 2 × TaqPCR MasterMix enzyme, and ddH_2_O to a final volume of 25 μL. The PCR amplification program was set as following: 95°C for 5 minutes for initial denaturation; 95°C for 30 seconds, followed by gradient annealing from 62°C to 52°C for 30 seconds, and extension at 72°C for 30 seconds, running for 10 cycles; 95°C for 30 seconds, 52°C for 30 seconds, and 72°C for 30 seconds, for 25 cycles; a final extension at 72°C for 20 minutes, and storage at 4°C. Following PCR, amplification products were analyzed using fluorescent capillary electrophoresis. GeneMarker software was used to analyze the results, including determining allele counts, peak patterns, and genotypes for each sample.

### 2.5. Data analysis

Phenotypic data were organized and analyzed using Excel 2019, calculating the distribution frequency, diversity index (H’), standard deviation, mean, and coefficient of variation (Coefficient of Variation(CV)=(Standard Deviation÷Mean)×100%). SPSS 22.0 was used to standardize all phenotypic data, followed by hierarchical clustering analysis, and the construction of a dendrogram using the system clustering method. The Genetic diversity index (H’) was calculated as: H′ = -ΣPiln Pi. Where Pi is the frequency of the i-th descriptive value of the trait. For quantitative traits, the Genetic diversity index (H’) was computed after qualitative treatment, assigning different grades to each observation following the method of Guo et al [14].

The SSR marker amplification results were read as “1” for a presence of a band at a given locus and “0” for absence, forming a 1/0 matrix. In GenAlEx version 6.501, SSR locus polymorphism was assessed, including the calculation of observed alleles (Na), effective alleles (Ne), Shannon ′ s information index (I), polymorphism information content (PIC), observed heterozygosity (Ho), and expected heterozygosity (He). Variation, differentiation, and genetic differentiation coefficient (Fst) as well as gene flow (Nm) among and within populations were calculated. Genetic distances between populations were computed using PowerMarker software. Clustering analysis was performed and a circular dendrogram was drawn with the UPGMA method. A Mantel test was performed using the skbio package in Python to assess the correlation between genetic distance (based on SSR markers) and phenotypic Euclidean distance. STRUCTURE 2.3.4 was used to analyze the population structure of 40 samples, with settings of K = 10, a burn-in period of 10,000, and MCMC (Markov Chain Monte Carlo) set to 100,000. Each K value was run 20 times, and the optimal ΔK value was calculated.

## 3. Result

### 3.1. Descriptive phenotypic Characterization

The statistical results of the distribution frequency, coefficient of variation (CV), and Shannon-weaver diversity index of the descriptive phenotypic traits of the tested tea resources were shown in Table 4. The coefficients of variation for the 18 descriptive traits ranged from 15.76% to 50.64% (all > 10%), with an average of 29.48%, indicating considerable variability among these traits, among which the coefficient of leaf color was the largest and tree type was the smallest. The coefficient of variation of 13 traits was higher than the average value, such as tree posture, branch density, bud and leaf pubescence, leaf color, leaf surface, leaf blade, serration sharpness, serration density, serration depth, leaf base, leaf tip, leaf margin and leaf size was higher than the average value, indicating that these 13 traits varied greatly in the tested tea resources. The Shannon-weaver diversity index ranged from 0.38 to 1.04, with an average of 0.76. The coefficient of serration sharpness was the largest and the tree type was the smallest. The diversity indexes of 12 traits were higher than the average value, such as branch density, bud leaf color, leaf shape, leaf color, leaf surface, leaf blade, leaf quality, serration sharpness, serration density, leaf tip, leaf margin and leaf size were higher than the average value, and the diversity indexes of leaf color, serration sharpness, serration density, leaf tip and leaf size were larger (≥1), indicating that the variation types of these 5 traits were rich.

**Table 4:** Coefficient of variation and Shannon-weaver index of non-numerical character.

traits	Description code and distribution frequency					Coefficient of variation/%	Shannon-weaver diversity index
	1	2	3	4	5		
Tree type	0.00	87.50	12.50	–	–	15.76	0.38
Tree posture	50.00	40.00	10.00	–	–	41.99	0.71
Branch density	62.50	25.00	12.50	–	–	47.74	0.90
Bud and leaf color	0.00	37.50	50.00	12.50	0.00	24.36	0.97
Bud and leaf pubescence	12.50	50.00	27.50	10.00	0.00	35.47	0.70
Leaf blade shape	0.00	5.00	7.50	62.50	25.00	17.91	0.83
Leaf blade color	37.50	37.50	12.50	12.50	–	50.64	1.00
Leaf surface relief	25.00	60.00	15.00	–	–	32.38	0.94
Leaf blade curvature	12.50	42.50	45.00	–	–	29.68	0.98
Leaf blade texture	12.20	62.50	25.00	–	–	28.57	0.90
Serration sharpness	25.00	50.00	25.00	–	–	35.81	1.04
Serration density	15.00	47.50	37.50	–	–	29.77	1.01
Serration depth	40.00	47.50	12.50	–	–	39.36	0.72
Leaf base shape	87.50	12.50	–	–	–	29.77	0.38
Leaf apex shape	10.00	40.00	35.00	15.00	–	34.34	1.02
Leaf margin undulation	40.00	47.50	12.50	–	–	38.28	0.98
Leaf attitude	12.50	75.00	12.50	0.00	–	25.32	0.74
Leaf size	25.00	62.50	12.50	0.00	–	32.38	1.00
Mean						29.48	0.76

### 3.2. Numerical phenotypic trait analysis

The results of numerical phenotypic traits of the tested tea resources were shown in Table 5, The leaf length of 40 tea resources ranged from 4.66 to 18.25, with an average of 10.42. The leaf width ranged from 1.78 to 6.57, with an average of 3.61. The maximum length-width ratio was 7.6, the minimum value was 1.17, and the average value is 3.12. The leaf area ranged from 7.93 to 69.68, with an average of 27.10. The coefficients of variation (CV) for the quantitative traits ranged from 31.91% to 56.86%, all exceeding 10%, with an average of 41.43%. This indicates considerable variability and relatively low consistency among these traits. The Shannon-weaver diversity index was the highest length-width ratio (1.93) and the smallest leaf area (1.70), with an average value of 1.86, and the figures of leaf width, leaf length and length-width ratio were higher than those of phenotypic traits. Overall, the numerical phenotypic traits of the 40 tested tea resources showed rich diversity.

**Table 5:** Variation of numerical phenotypic traits.

Material name	Leaf length	Leaf width	Length-width ratio	Leaf area
S1	15.81 ± 0.86	5.49 ± 0.74	2.91 ± 0.40	60.74 ± 8.61
S2	16.99 ± 1.10	5.31 ± 0.60	3.23 ± 0.45	63.00 ± 6.78
S3	16.16 ± 1.04	5.56 ± 0.59	2.93 ± 0.42	62.79 ± 5.88
S4	16.82 ± 0.85	5.25 ± 0.97	3.29 ± 0.74	61.46 ± 8.59
S5	16.72 ± 1.72	5.49 ± 0.95	3.14 ± 0.79	63.49 ± 4.08
S6	7.87 ± 1.06	3.82 ± 1.02	2.13 ± 0.44	21.29 ± 6.95
S7	7.67 ± 1.23	3.86 ± 0.99	2.08 ± 0.68	20.63 ± 4.48
S8	7.68 ± 0.73	2.26 ± 0.63	1.84 ± 0.40	22.71 ± 1.75
S9	7.47 ± 1.26	4.25 ± 1.10	1.89 ± 0.81	21.86 ± 5.20
S10	7.89 ± 0.82	4.07 ± 0.90	1.99 ± 0.45	22.62 ± 6.55
S11	12.57 ± 1.20	2.82 ± 1.02	5.00 ± 2.28	24.26 ± 6.57
S12	13.13 ± 0.86	2.51 ± 0.63	5.53 ± 1.79	22.97 ± 5.74
S13	13.20 ± 1.02	3.03 ± 0.75	4.57 ± 1.30	27.68 ± 4.49
S14	12.06 ± 0.88	3.04 ± 0.92	4.26 ± 1.49	25.47 ± 7.26
S15	12.35 ± 1.00	3.26 ± 0.63	3.87 ± 0.69	28.30 ± 6.91
S16	12.64 ± 0.86	3.05 ± 0.82	4.29 ± 0.88	27.27 ± 8.91
S17	12.38 ± 1.49	3.00 ± 0.93	4.44 ± 1.73	25..70 ± 7.11
S18	12.88 ± 1.10	3.62 ± 0.85	3.59 ± 0.31	32.94 ± 7.87
S19	11.41 ± 0.82	3.83 ± 1.11	3.13 ± 0.79	30.77 ± 9.72
S20	14.28 ± 1.16	3.07 ± 0.80	4.90 ± 1.55	30.59 ± 7.92
S21	6.60 ± 1.14	2.77 ± 0.82	2.57 ± 1.09	12.75 ± 4.50
S22	6.96 ± 1.06	3.34 ± 0.50	2.11 ± 0.41	16.30 ± 3.57
S23	7.59 ± 0.63	2.91 ± 1.13	2.95 ± 1.33	15.14 ± 4.66
S24	6.94 ± 0.91	2.80 ± 1.22	2.82 ± 1.19	13.22 ± 4.29
S25	7.64 ± 0.74	2.77 ± 0.90	2.90 ± 0.66	15.02 ± 6.01
S26	12.38 ± 0.94	3.15 ± 0.92	4.16 ± 1.28	27.35 ± 8.88
S27	11.89 ± 0.94	3.40 ± 1.15	3.78 ± 1.27	27.96 ± 7.81
S28	12.58 ± 1.11	3.39 ± 0.62	3.78 ± 0.71	29.93 ± 6.33
S29	11.88 ± 0.86	3.09 ± 0.78	4.02 ± 1.07	25.60 ± 6.29
S30	13.35 ± 1.22	2.69 ± 0.48	5.11 ± 1.33	24.97 ± 3.84
S31	5.97 ± 0.73	2.890.96±	2.16 ± 0.40	12.39 ± 5.77
S32	6.47 ± 0.83	2.65 ± 0.66	2.53 ± 0.64	12.08 ± 3.80
S33	5.65 ± 0.89	2.75 ± 0.65	2.10 ± 0.39	11.06 ± 4.09
S34	5.96 ± 0.86	0.94 ± 1.32	2.32 ± 1.09	12.08 ± 5.12
S35	6.84 ± 1.04	2.61 ± 0.79	2.82 ± 1.03	12.20 ± 2.18
S36	7.91 ± 0.94	4.50 ± 1.22	1.87 ± 0.61	24.42 ± 3.74
S37	7.61 ± 0.61	4.38 ± 0.64	1.77 ± 0.37	23.15 ± 1.49
S38	8.09 ± 1.33	4.52 ± 1.12	1.89 ± 0.60	25.13 ± 3.66
S39	8.36 ± 1.04	4.43 ± 1.07	2.00 ± 0.71	25.70 ± 6.13
S40	8.20 ± 1.25	3.75 ± 0.57	2.26 ± 0.71	21.19 ± 0.95
Minimum value	4.66	1.78	1.17	7.93
Maximum value	18.25	6.57	7.60	69.68
Mean value	10.42	3.61	3.12	27.10
standard deviation	3.56	1.15	1.34	15.41
coefficient of variation/%	34.14	31.91	42.81	56.86
Shannon-weaver diversity index	1.91	1.90	1.93	1.70

### 3.3. Cluster analysis of phenotypic traits

**Fig 1:**
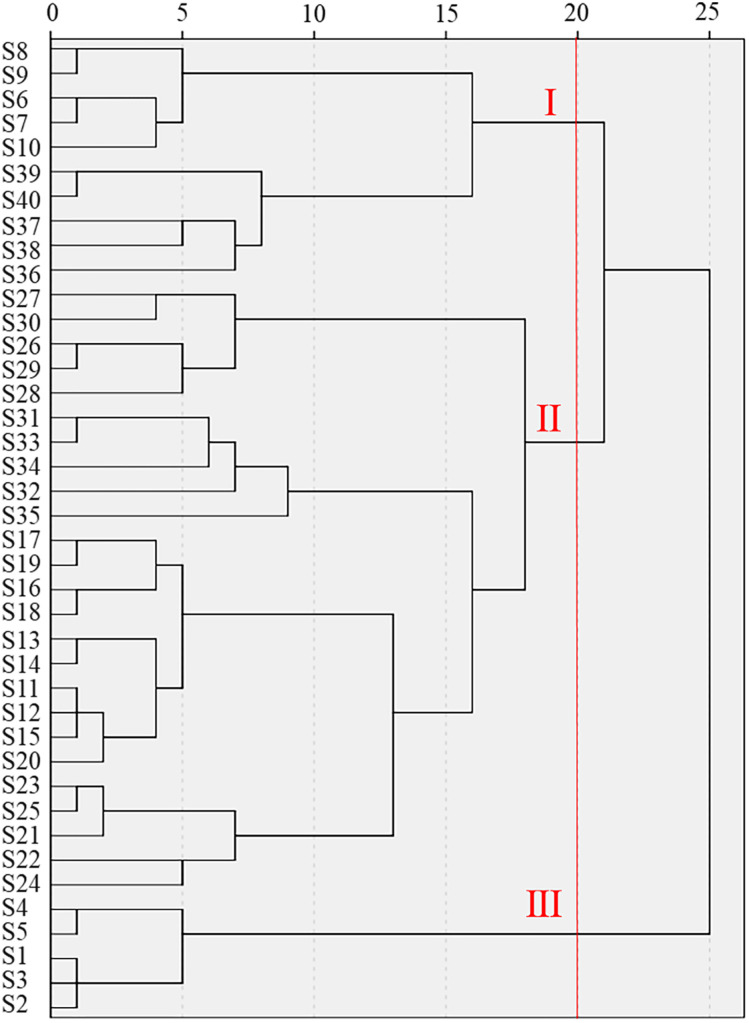
Cluster analysis of 40 samples of wild tea plants resources based on phenotypic traits.

The cluster analysis was carried out based on the Euclidean genetic distance by systematic clustering method (Fig 1). The results showed that when the Euclidean distance was 20, the 40 tea plants samples were divided into three groups. Group I contained 10 resources, including 5 resources of the Raoxiu population (RS) and 5 resources of the Jiangbian population (JB). The principal tree type of Group I was small tree type, with the morphology characteristics of medium leaf size, erect growth habit, dense branching, yellow-green bud leaves, flat leaf surface, flat leaf surface, hard leaf texture, obtuse leaf serrations, flat leaf margin, and semi-erect leaf attitude. Group Ⅱ contained 25 resources, Group II contained 25 resources, including 10 resources from Changqiao population (CQ), 5 resources from Xinlin population (XL), 5 resources from Liutou population (LT) and 5 resources from Nanei population (NN). The principal tree type of Group II was small tree type, with the morphological characteristics of moderate to dense branching density, yellow-green to pale green bud leaves, oblong to lanceolate leaf shape, slightly raised to raised leaf surface, cuneate leaf base, ascending to slightly ascending leaf insertion angle, and small to medium leaf size. Group Ⅲ contained 5 resources, all from Qingshan population (QS). The principal morphological characteristics include: arboreous habit, large leaf size, sparse branching density, green bud leaves, sparsely pubescent of buds and leaf, oblong-elliptic leaf shape, dark green leaf color, slightly raised leaf surface, slightly recurved leaf blade, medium leaf blade texture, sharp and dense leaf serrations, cuneate leaf base, obtuse leaf apex, undulate leaf margin, and horizontal leaf attitude. Group III was distinguished from Group I and II primarily by tree-structured, branch density, bud leaf coloration, leaf color, leaf insertion angle, and leaf size. Group I and II were differentiated mainly by bud leaf coloration, leaf surface morphology, leaf quality, and serration acuteness.

### 3.4. Polymorphism analysis of SSR loci

The polymorphism of 40 tea samples was detected by 14 pairs of primers (Table 6). A total of 117 alleles (Na) were detected totally, with an average number of 8.36 alleles. Among these data, the number of alleles detected at A5 and CSK065 was the least, which was 4, and the number of alleles detected at CsFM1715 was the most, which was 15. A total of 58.86 effective number of alleles (Ne) were detected at 14 loci, ranging from 1.36 to 8.41, with an average of 4.20, accounting for 50.4%. The Shannon diversity index (I) ranged from 0.54 to 2.36, with a mean value of 1.56. The observed heterozygosity (Ho) ranged from 0.08 to 0.87, with an average of 0.57. The expected heterozygosity (He) ranged from 0.26 to 0.88, with an average of 0.69. Among the 14 loci, 13 loci of the expected heterozygosity (He) were greater than the observed heterozygosity (Ho), and the observed heterozygosity (Ho) of 1 locus was greater than the expected heterozygosity (He). The mean value of fixation index (F) is 0.19. The polymorphism information content (PIC) ranged from 0.25 to 0.87, with an average of 0.66. Among them, there were 12 loci with PIC greater than 0.5, 1 locus with PIC between 0.25 and 0.5, and 1 locus with PIC less than 0.25. The SSR primers selected by the research had a high level of amplification site polymorphism on the tested materials, and the polymorphism of different SSR loci was significantly different, indicating that the 40 ancient tea tree resources tested had rich genetic diversity.

**Table 6:** Polymorphism of amplification products by SSR primer of tea populations in Chongzuo.

Primer name	The number of alleles (Na)	Effective number of alleles(Ne)	Shannon information index(I)	Observed heterozygosity (Ho)	Expected heterozygosity (He)	Fixation index(F)	Polymorphic information content (PIC)
234	8	3.76	1.58	0.70	0.73	0.05	0.69
478	5	3.75	1.39	0.40	0.73	0.46	0.69
A5	4	1.58	0.72	0.44	0.37	−0.20	0.34
CsFM1051	9	5.45	1.89	0.76	0.82	0.07	0.79
CsFM1058	8	4.74	1.71	0.69	0.79	0.12	0.76
CsFM1068	14	8.41	2.31	0.74	0.88	0.16	0.87
CsFM1158	6	3.19	1.32	0.45	0.69	0.35	0.64
CsFM1599	5	3.78	1.42	0.64	0.74	0.13	0.69
CsFM1696	10	4.34	1.83	0.65	0.77	0.16	0.75
CsFM1715	15	8.28	2.36	0.88	0.88	0.01	0.87
CsFM1828	13	5.00	2.05	0.74	0.80	0.07	0.79
CSK014	9	2.62	1.33	0.44	0.62	0.30	0.58
CSK065	4	1.36	0.54	0.08	0.26	0.69	0.25
SSR1	7	2.61	1.36	0.44	0.62	0.29	0.59
Mean	8.36	4.20	1.56	0.57	0.69	0.19	0.66

### 3.5. Population genetic diversity analysis

The results of genetic diversity of the seven populations were shown in Table 7. The number of alleles (Na) ranged from 2.29 to 5.50, with an average of 3.21. The effective number of alleles (Ne) ranged from 1.81 to 3.71, with an average of 2.51. The proportion of effective alleles ranged from 67.36% to 88.35%, and the alleles were evenly distributed in each population. The observed heterozygosity (Ho) of the population ranged from 0.45 to 0.70, with an average of 0.56. The expected heterozygosity (He) ranged from 0.36 to 0.65, with an average of 0.50. Shannon diversity index (I) ranged from 0.60 to 1.34, with an average of 0.89. In conclusion, these results demonstrate that all seven populations possess rich genetic diversity.

**Table 7:** Genetic diversity parameters among 7 populations of tea trees in Chongzuo.

Population code	The number of alleles (Na)	Effective number of alleles (Ne)	Percentage of effective alleles (%)	Shannon information index (I)	Observed heterozygosity (Ho)	Expected heterozygosity (He)
CQ	5.50	3.71	67.36	1.34	0.64	0.65
LT	2.43	2.15	88.35	0.74	0.53	0.46
JB	3.57	2.94	82.33	1.07	0.70	0.60
NN	2.64	2.04	77.26	0.70	0.51	0.40
RX	3.50	2.79	79.60	1.00	0.59	0.54
QS	2.29	1.81	79.05	0.60	0.45	0.36
XL	2.57	2.13	82.69	0.76	0.53	0.46

### 3.6. Genetic structure analysis

**Fig 2:**
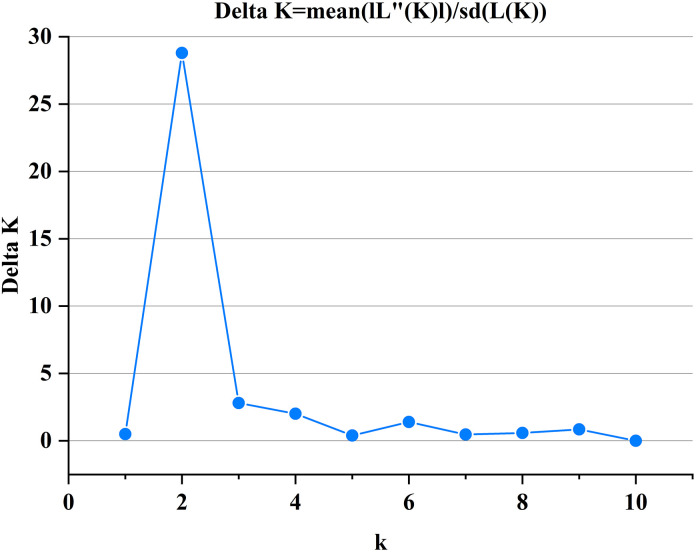
Relationship between K and ΔK.

**Fig 3:**
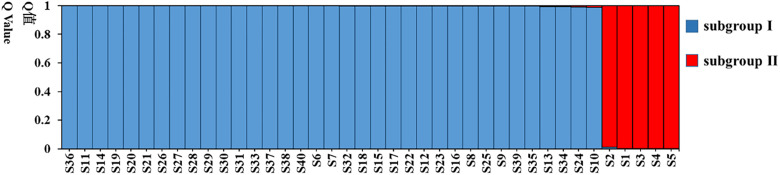
Model-based on population structure analysis of 40 evaluated germplasms (K = 2). Numbers of S1 to S40 are shown in Table 1.

The genetic structure analysis of 40 tea resources showed that ΔK was the largest when it is at K = 2 (Fig 2). The 40 tea plant resources could be divided into two subgroups (Fig 3). There were 35 resources in subgroup I (blue) and 5 resources in subgroup II (red). Among them, 5 tea plant samples (S1-S5) of QS were distributed in subgroup II, and 35 tea plant samples (S6-S40) of the other 6 populations were distributed in subgroup I. According to the Q value distribution of the two subgroups, among the 40 tested tea tree resources, the Q value of S6-S40 was > 0.9, and the Q value of S1-S5 was < 0.6, indicating that the genetic background of QS was complex.

### 3.7. Analysis of the population genetic relationship

**Fig 4:**
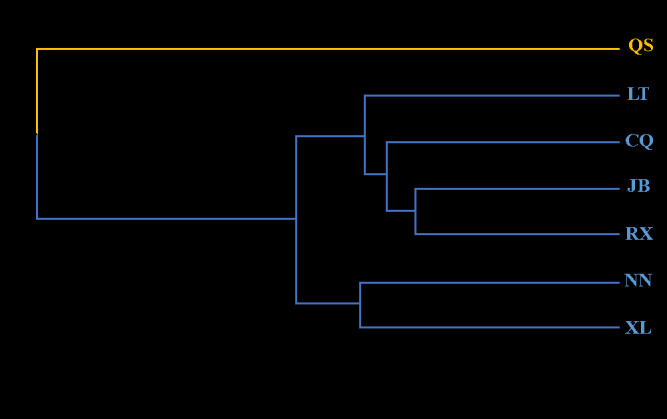
Genetic distance clustering among 7 populations of tea trees in Chongzuo.

The genetic distance among the seven tea populations ranged from 0.24 to 0.74 (Table 8). Among them, the genetic distance of JB and RX was the shortest, and the genetic distance of QS and LT was the longest. The genetic distance clustering among the seven tea tree populations showed that (Fig 4), when the genetic distance was 0.20, it was clustered into two categories. Among them, the QS was clustered into group I alone, and the CQ, LT, JB, RX, NN and XL were clustered into group II. The QS had the farthest genetic relationship with other native populations. At the genetic distance of 0.17, the six populations in group II were clustered into two groups. Among the six populations, NN and XL were clustered into one group, and CQ, LT, JB and RX were clustered into the other group. NN and XL are distantly related to CQ, LT, JB, and RX populations. At the genetic distance of 0.12, only JB and RX were clustered together, and the genetic relationship was the closest. The population genetic relationship showed that the QS was far from the other six populations.

**Table 8:** Genetic distance among 7 populations of tea plants in Chongzuo.

Populations code	CQ	LT	JB	NN	RX	QS
LT	0.32					
JB	0.25	0.25				
NN	0.35	0.41	0.34			
RX	0.30	0.33	0.24	0.45		
QS	0.68	0.74	0.70	0.69	0.65	
XL	0.30	0.41	0.34	0.30	0.45	0.66

### 3.8. Analysis of genetic relationship of single tea plant

**Fig 5:**
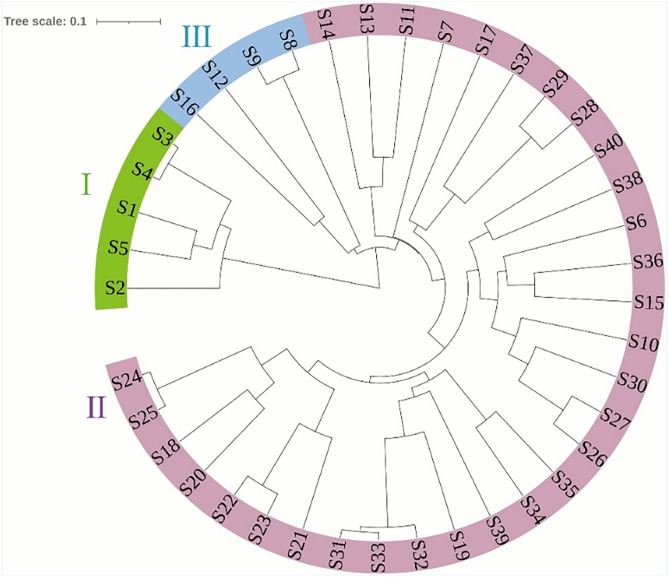
UPGMA cluster of 40 germplasm resources of Chongzuo tea trees based on SSR markers.

The genetic distance among the 40 tea resources ranged from 0.20 to 0.95 (Fig 5). When the genetic distance was 0.33, 40 tea samples were divided into three groups. The five resources of the QS were clustered into the first category, accounting for 12.5% of the survey resources. The five resources of QS were clustered into group I, accounting for 12.5% of the tested resources. Group II had 31 tea plant resources, accounting for 77.5% of the tested resources. Among them, tea resources from different regions were clustered into different subgroups according to genetic distance, and the subgroups were not clustered according to the same geographical location. Group III contained 4 tea plant resources, accounting for 10.00% of the survey resources, which were S8, S9 of RX and S12, S16 of CQ. The single tea plant genetic relationship showed that the genetic relationship among the 5 resources of the QS and the other 35 resources was distant, which is consistent with the results of the population genetic relationship analysis.

To evaluate the relationship between clustering based on phenotypic traits and SSR markers, a Mantel test was conducted between the genetic distance matrix (SSR markers) and the Euclidean distance matrix (phenotypic traits). A significant correlation was found (r = 0.211, *P* = 0.003 < 0.05), indicating that phenotypic divergence among the 40 tea plant samples is associated with their genetic differentiation.

### 3.9. Population genetic differentiation and gene flow analysis

The results of genetic differentiation coefficient (Fst) and gene flow (Nm) of 7 tea populations were shown in Table 9. The range of Fst among populations was 0.06–0.38. The Fst value between QS and NN was the largest, and the Fst value between QS and LT, XL, JB, RX, CQ were greater than 0.25. The Fst values between NN and (LT, JB, RX, XL), between XL and (LT, RX), and between RX and LT ranged from 0.15 to 0.25. The Fst values among other populations were less than 0.15. The gene flow (Nm) ranged from 0.41 to 4.06. The Nm between QS and LT, XL, JB, RX, CQ, NN was less than 1, while it was greater than 4 between CQ and JB, and the Nm of other groups was in the range of 1–4. The results of population genetic differentiation and gene flow showed that the genetic differentiation between QS and the other six populations was greater, and gene flow was lower.

**Table 9:** Genetic differentiation coefficient (Fst) (below diagonal) and gene flow (Nm) (above diagonal) among the seven tea populations in Chongzuo.

Population code	CQ	LT	JB	NN	RX	QS	XL
CQ		1.98	4.06	1.69	2.69	0.71	2.53
LT	0.11		2.30	0.86	1.34	0.42	1.17
JB	0.06	0.10		1.35	2.69	0.57	1.83
NN	0.13	0.23	0.16		1.09	0.41	1.41
RX	0.09	0.16	0.09	0.19		0.68	1.18
QS	0.26	0.37	0.31	0.38	0.27		0.52
XL	0.09	0.18	0.12	0.15	0.18	0.32	

### 3.10 analysis of molecular variance among populations

The analysis of molecular variance among 7 populations was shown in Table 10. The genetic variation among individuals was 74.22%, the genetic variation among populations was 20.46%, and the genetic variation among individuals within populations was 5.32%. The genetic variation of the tested materials mainly came from populations and individuals.

**Table 10:** Analysis of molecular variance for intra-and inter-population variations.

Source of variation	degree of freedom	Sum of squares	mean square	variance component	Percentage of variation (%)
Among population	6	99.29	16.55	1.08	20.46%
Among individuals within populations	33	147.15	4.46	0.28	5.32%
Among individuals	40	156	3.90	3.90	74.22%
Total	79	402.44		5.26	100%

## 4. Discussion

Guangxi wild tea plants are widely distributed with unique local characteristics [15,16]. Studies have demonstrated the high genetic diversity and significant utilization value in several wild tea plant resources from Guangxi, including Jinxiu wild tea [17], the Sanjiang tea plant population [18], and wild tea resources from Liubao Town in Wuzhou, Guangxi [19]. After long-term natural selection or artificial domestication, wild tea plants showed diversity and stability variation from molecular level to phenotypic traits, which laid a foundation for the screening of tea plant germplasm resources and genetic breeding research [20].

Plant phenotypic traits are affected by the interaction of plant genotype and growth environment. The description and identification of traits are the most basic methods and approaches for germplasm resources research. The coefficient of variation and diversity index are important indicators to reflect the genetic diversity of crops, and are important basis for distinguishing different populations of tea plants [21]. In this study, the diversity of descriptive phenotypic traits and numerical phenotypic traits of 40 tea plant samples were analyzed. The coefficient of variation of 18 descriptive phenotypic traits ranged from 15.76% to 50.64%, with an average of 29.48%, all of which exceeded 10% [22], indicating that these descriptive traits had poor consistency and were rich in variation. The diversity index of descriptive phenotypic traits ranged from 0.38 to 1.04, and the mean value was 0.76. It was higher than the diversity index of descriptive phenotypic traits reported by 195 arbor tea trees in Guizhou (0.67) [8], Sandu wild tea (0.26) [23], and large tea plants in central Guizhou (0.42) [24]. The coefficient of variation of numerical phenotypic traits ranged from 0.32 to 0.57, indicating that these numerical phenotypic traits had poor consistency and were rich in variation, which was higher than that of Gaolu tea population [25]. The diversity index of numerical phenotypic traits ranged from 1.70 to 1.93, with an average of 1.86, which was higher than 1 (the average value of tea resources in China was 0.96) [8]. It indicated that the phenotypic traits of the tested tea plants resources were rich in variation. Cluster analysis of phenotypic traits showed that the materials in each group could be distinguished by population, indicating that the phenotypic traits of each group were significantly different. The three groups were distinguished by tree-structured, branch density, bud leaf coloration, leaf color, leaf insertion angle and leaf size, leaf surface morphology, leaf quality and serration acuteness, indicating that these nine phenotypes were the main factors for the differences in phenotypic traits of the tested tea resources.

In this study, the average number of alleles (Na) detected by 14 pairs of SSR primers was 8.36, the average number of effective alleles (Ne) was 4.20, the average Shannon diversity index (I) was 1.56, and the average polymorphism information content (PIC) was 0.66. It is higher than the mean values of Na, Ne and PIC reported in the analysis of genetic diversity of Shiqian tea resources based on SSR molecular markers [26]. It is higher than the average value of Na, Ne and I reported in a study on the genetic diversity and population structure of wild tea germplasm from Badong County, Hubei Province [27]. The above results indicate that the 14 pairs of SSR primers screened in this study are highly polymorphic and that the alleles are evenly distributed within the population, making them suitable for subsequent research.

The genetic diversity analysis of 7 tea plant populations in Chongzuo using 14 pairs of SSR primers showed that the mean value of Shannon diversity index (I) was 0.89. The I value is a parameter that measure the level of genetic diversity within a population. Higer values of I indicate increased population diversity and richer genetic diversity [28]. The average Shannon diversity index in this study was higher than the average Shannon diversity index of 122 Yunnan wild tea plants (0.88) [29], and of 38 Huangjincha individuals (0.55) [30]. According to the concept of heterozygosity, when the heterozygosity is greater than 0.5, the population has relatively rich genetic diversity, and the closer the observed heterozygosity value is to the expected heterozygosity value, the higher the genetic diversity of the population [31,32]. The mean values of observed heterozygosity and expected heterozygosity in this study were 0.56 and 0.50 respectively, which were relatively close to each other, higher than the mean values of observed heterozygosity (0.32) and expected heterozygosity (0.28) of Anxi tea resources in Fujian [33], and also higher than the mean values of observed heterozygosity (0.37) and expected heterozygosity (0.30) of Wuyishan tea resources [34]. The results of I and heterozygosity in this study indicate that the seven tea tree populations in Chongzuo possess high genetic diversity.

The genetic structure of a population is defined by the composition of its subgroups, which in turn reveals the distinctive characteristics of those subgroups. In this study, ΔK is the largest when K is 2, indicating that the optimal population number is 2. According to the method of Evanno et al. [35], the 40 analyzed tea plant samples were clustered into two subgroups. Five materials in subgroup II were all from QS, and the Q value was less than 0.6. The research showed that when the Q-value of a test material in the subgroup exceeded 0.6, it suggested a relatively simple genetic background; conversely, a lower Q-value corresponded to a more complex genetic relationship [36]. The results of this study showed that the genetic background of QS was complex.

Analysis of the genetic relationships estimated that the genetic distance among the seven tea tree populations ranged from 0.24 to 0.74. When the genetic distance was 0.20, the QS was clustered into one group alone, and there was a clear boundary between the group of QS and the group of other six populations. The genetic distance among the 40 tea plant individuals ranged from 0.20 to 0.95, which was greater than that among populations, indicating substantial genetic differences among individuals. When the individual genetic distance was 0.33, the 40 samples were divided into three groups, and five samples from the QS were clustered into one group. The genetic distance clustering between populations and individuals showed that the QS and the five samples contained had specific genetic background, which may be due to long-term natural selection and certain genetic variation. QS can be used as one of the key materials for screening excellent tea resources.

Genetic drift, mating system, and gene flow are important factors affecting genetic structure. Among them, gene flow can counteract genetic drift and reduce genetic differentiation [37]. It has been reported that the genetic differentiation coefficient (Fst) reflects the degree of population differentiation, with a larger Fst value indicating a higher level of differentiation [38]. Gene flow (Nm) can measure the level of gene flow among populations. Generally, when Nm is greater than 4, the gene flow among populations is sufficient. If Nm is less than 1, it is less [39]. In this study, the (Fst) among the QS and the other six tea populations was greater than 0.25 and the Nm was less than 1. It is speculated that the QS may be due to the increase of genetic differentiation caused by genetic drift, resulting in a high degree of genetic differentiation among the QS and the other six tea populations. There was a certain degree of gene flow among the other 6 populations (except QS), and the degree of genetic differentiation was not high. This was consistent with the results of molecular variance analysis between populations (the genetic variation between populations was 20.46%, and the variation between individuals was 74.23%). The tea plant populations in different regions are affected by natural selection, artificial selection, geographical environment and distribution area, which will cause the change of population genetic structure, the gradual increase of genetic difference and the formation of certain genetic differentiation. The comparative analysis of genetic structure and genetic differentiation of tea plant samples in Guangdong and Guangxi showed that the degree of genetic differentiation was large due to natural environmental factors [40]. In this study, the altitude of QS was 716.53 meters, which was higher than that of other tea populations. Therefore, it was speculated that the altitude difference was one of the reasons for the high genetic differentiation of this population.

In this study, both the population-based and individual-based kinship clustering analyses, derived from phenotypic clustering and SSR marker-based population genetic structure, successfully distinguished the five tea plants resources from the Qingshan (QS) population. Furthermore, the Mantel test indicated a significant correlation between phenotypic and genetic distances, suggesting that the five QS resources possess distinct phenotypic characteristics and a complex genetic background. Except for the five resources of QS, the consistency of phenotypic clustering and SSR marker clustering results of other resources was poor, which has been reported in other crops [41,42]. The phenotype observed in this study is only a part of the phenotype of wild tea resources, and the SSR marker represents a difference in DNA, and this genetic difference has little or no correlation with phenotype. On the other hand, most phenotypic traits are determined by multiple genes. The number of SSR primers in this study is limited and cannot represent all the DNA information of the observed phenotypic traits.

## 5. Conclusions

The genetic differences among wild tea resources in Chongzuo area of Guangxi are large, the genetic basis is wide, and the genetic diversity is rich, especially the genetic background of QS. This result provides a reference for further excavation and utilization of Chongzuo wild tea germplasm resources, cultivation of new tea varieties and development of new tea products in the future. In addition, in order to avoid the loss of characteristics due to the mixed evolution among different tea plant populations, key areas should be selected or resource nurseries should be established to protect the wild tea plants germplasm resources in Chongzuo.

## Supporting information

S1 File Raw data. (ZIP)
